# Nichtmedikamentöse Therapiemaßnahmen, Rehabilitationsleistungen und Mitgliedschaft in Selbsthilfeorganisationen bei axialer Spondyloarthritis (Die ATTENTUS axSpA-Studie)

**DOI:** 10.1007/s00393-023-01410-w

**Published:** 2023-09-19

**Authors:** D. Meyer-Olson, K. Hoeper, L. Hammel, S. Lieb, A. Haehle, U. Kiltz

**Affiliations:** 1 Klinik für Rheumatologie und Immunologie, Medizinische Hochschule Hannover, Carl-Neuberg-Str. 1, 30625 Hannover, Deutschland; 2 Rheumatologie und Immunologie, m&i Fachklinik Bad Pyrmont/MVZ Weserbergland, Bad Pyrmont, Deutschland; 3 Regionales Kooperatives Rheumazentrum Niedersachsen e. V., Hannover, Deutschland; 4 Deutsche Vereinigung Morbus Bechterew e. V., Schweinfurt, Deutschland; 5 Novartis Pharma GmbH, Nürnberg, Deutschland; 6 Rheumazentrum Ruhrgebiet, Herne, Deutschland; 7 Ruhr Universität, Bochum, Deutschland

**Keywords:** Spondylarthropathien, Patientengruppen, Physikalische Therapieformen, Hilfsmittel, Bewegungstherapien, Spondylarthropathies, Self-Help Groups, Physical Therapy Modalities, Assistive devices, Exercise Training

## Abstract

**Hintergrund:**

Die Behandlung der axialen Spondyloarthritis (axSpA) umfasst neben medikamentösen Therapiemaßnahmen (MTM) auch nichtmedikamentöse Therapiemaßnahmen (NMTM) sowie unterstützende Ressourcen wie rehabilitationsmedizinische Therapieleistungen (RTL) und die Mitgliedschaft in Selbsthilfeorganisationen (SHO). Trotzdem bestehen deutliche Teilhabeeinschränkungen bei Patient*innen mit axSpA in Deutschland.

**Ziel der Arbeit/Fragestellung:**

Untersuchung der Funktions- und Teilhabeeinschränkungen und Nutzung von MTM, NMTM, RTL und SHO bei Patient*innen mit axSpA.

**Material und Methoden:**

Multizentrische, deutschlandweite Beobachtungsstudie von 770 axSpA-Patient*innen (ATTENTUS-axSpA).

**Ergebnisse:**

Es bestehen deutliche Funktions- und Teilhabeeinschränkungen bei den axSpA-Patient*innen; 39 % erhielten keine Therapie mit biologischen krankheitsmodifizierenden Medikamenten (bDMARDs). Bei den NMTM wurden bewegungstherapeutische Maßnahmen bei 54 % weniger als 1‑mal die Woche und bei 29 % 1‑mal pro Woche verordnet. Eine regelmäßige Bewegung führten 86 % der Patient*innen durch, hauptsächlich in Form häuslicher Übungen. Training im Fitnessstudio (14 %) oder Vereinssport (7 %) wurden seltener ausgeführt. Eine RTL erhielten 54 % der Patient*innen, und bei etwa einem Drittel lag die letzte RTL über 5 Jahre zurück. Es waren 13 % Mitglieder in einer SHO. In dieser Gruppe fand sich eine signifikant höhere Inanspruchnahme von NMTM und RTL.

**Diskussion:**

Verfügbare Behandlungsoptionen und Ressourcen werden von axSpA-Patient*innen häufig in geringem Maß und/oder in niedriger Intensität genutzt, welches eine mögliche Erklärung für persistierende Teilhabeeinschränkungen sein könnte. Wir beobachteten eine verstärkte Inanspruchnahme von NMTM und RTL bei Mitgliedschaft in einer SHO.

## Abstract (en)

**Background:**

The treatment of axial spondylarthritis (axSpA) includes pharmacological treatment measures (PTM) and nonpharmacological treatment measures (NPTM) as well as supporting resources, such as rehabilitation services (RS) and membership in patient support groups (PSG). Nevertheless, there are significant participation restrictions in patients with axSpA in Germany.

**Objective:**

Investigation of functional deficits, participation restrictions and utilization of PTM, NPTM, RS and PSG membership in patients with axSpA.

**Material and methods:**

Multicentric, observational study of 770 axSpA patients in Germany (ATTENTUS-axSpA).

**Results:**

Substantial functional deficits and participation restrictions were observed in axSpA patients. Of the patients 39% did not receive treatment with biological disease-modifying antirheumatic drugs (bDMARD). In the NPTM 54% received physiotherapy less than once per week and 29% once per week. Physical activities were regularly performed by 86% of patients, mainly in the form of home exercises. Training in a gym (14%) or sports club (7%) was carried out much less frequently. Of the patients 54% received RS, one third had the last rehabilitation more than 5 years ago and 13% of the patients were members in a PSG. A significantly higher utilization of NPTM and rehabilitation was found in this group.

**Conclusion:**

Treatment options and resources were often utilized to a small extent and/or in low intensity by axSpA patients, which could be a possible explanation for persisting restrictions of participation. Membership in a PSG was associated with an increased utilization of NPTM and RS.

## Hintergrund

Die axiale Spondyloarthritis (axSpA) ist eine entzündlich rheumatische Systemerkrankung des Achsenskeletts, welche mit deutlichen Lebensqualitätseinschränkungen assoziiert ist [1]. Die leitliniengestützten Therapieoptionen der axSpA umfassen medikamentöse (MTM) und nichtmedikamentöse Therapiemaßnahmen (NMTM) [2–6]. Zu den MTM zählen nichtsteroidale Antirheumatika (NSARs), Tumor-Nekrose-Faktor-Inhibitoren, Interleukin-17-Inhibitoren und Januskinaseinhibitoren [3]. Die MTM dienen v. a. zur Unterdrückung der Entzündungsaktivität und damit zur Verhinderung der Entstehung von strukturellen Schäden [1–3]. In Ergänzung dazu haben NMTM vorrangig eine Verbesserung oder Vermeidung von Bewegungseinschränkungen zum Ziel [2–5].

Nationale und internationale Leitlinien empfehlen eine Kombination aus MTM und NMTM [3]. Die regelmäßige Erfassung der körperlichen Funktionsfähigkeit z. B. mit dem Bath Ankylosing Spondylitis Functional Index (BASFI) und die Verordnung von NMTM bei eingeschränkter körperlicher Funktionsfähigkeit sind Teil der *Assessment of SpondyloArthritis International Society*(ASAS)-Qualitätsstandards für die axSpA, die inzwischen auch auf die Versorgungssituation in Deutschland adaptiert worden sind [7, 8].

Komplexere Aktivitäten der Selbstversorgung und des häuslichen Lebens werden mittels des validierten *Health Assessment Questionnaire Disability Index*(HAQ-DI)-Fragebogen [9] untersucht und eignen sich zur Einschätzung von Teilhabeeinschränkungen bei verschiedenen entzündlich rheumatischen Systemerkrankungen wie der axSpA [10]. Darüber hinaus wird der Einsatz von Hilfsmitteln ebenfalls im HAQ-DI erfasst.

Bei Einschränkungen der beruflichen oder sozialen Teilhabe stehen Betroffenen in Deutschland, gesetzlich verankert, ergänzende Ressourcen wie rehabilitationsmedizinische Therapieleistungen (RTL) zur Verfügung [11]. Im Rahmen einer RTL werden, abhängig vom aktuellen Bedarf der Patient*innen, MTM und NMTM in einem multimodalen Therapieansatz eingesetzt. Wir konnten kürzlich zeigen, dass die berufliche Teilhabe bei axSpA-Patient*innen in Deutschland in allen Dimensionen deutlich eingeschränkt ist [12]. Dieses Ergebnis war angesichts wesentlich verbesserter Behandlungsstrategien in den letzten Jahren und der vorhandenen Ressourcen, namentlich der Möglichkeit einer Inanspruchnahme von RTL, überraschend.

Als eine weitere Möglichkeit zur Verbesserung der Selbstmanagementstrategien von Patient*innen wird in Leitlinien die Anbindung an Selbsthilfeorganisationen (SHO) empfohlen [6]. Diese helfen, die Erkrankung besser zu verstehen und auch bessere Informationen über Behandlungsoptionen zu erlangen [13, 14].

In der aktuellen Untersuchung erfolgte eine Vertiefung dieser Auswertung, um erstens die Einschränkungen der Teilhabe detaillierter zu beleuchten und zweitens zu untersuchen, inwieweit MTM, NMTM, RTL und Mitgliedschaft in einer SHO von Patient*innen mit axSpA genutzt werden. Eine weitere wichtige Fragestellung war, ob die Mitgliedschaft in einer SHO mit einer verstärkten Nutzung von NMTM und RTL assoziiert war.

## Methode

### Studiendesign

Die ATTENTUS-axSpA-Studie war eine multizentrische, deutschlandweite Beobachtungsstudie, welche von November 2019 bis Juli 2020 durchgeführt wurde. Es wurden demografische, klinische und patientenberichtete Daten (Patient Reported Outcomes [PROs]) von Patient*innen mit einer ärztlich bestätigten axSpA-Diagnose (gemäß ICD-10 Code M45) erhoben. Eine ausführliche Beschreibung des Studiendesigns, des Studienablaufes und der Datenerhebung wurde kürzlich publiziert [12]. Von den 797 in die Studie aufgenommenen Patient*innen wurden 481 Patient*innen (60,4 %) von rheumatologischen Facharztpraxen und 251 Patient*innen (31,5 %) von Klinik‑/Universitätsambulanzen eingeschlossen. Weiterhin wurden die Nutzung von MTM, NMTM, RTL und die Mitgliedschaft in einer SHO erfragt. Als PROs wurden die Krankheitsaktivität mittels *Bath Ankylosing Spondylitis Disease Activity Index* (BASDAI) [15] und die körperliche Funktionsfähigkeit mittels BASFI [16] untersucht. Teilhabeeinschränkungen in den Bereichen Selbstversorgung und häusliches Leben sowie Hilfsmittelversorgung wurden mittels des HAQ-DI erhoben [9]. Die globale Funktionsfähigkeit und Gesundheit von axSpA-Patienten wurden anhand des *Assessment of SpondyloArthritis International Society Health Index *(ASAS-HI) bewertet [17]. Arbeitsproduktivität und Aktivität wurden über den *Work Productivity and Activity Impairment* (WPAI): *Spondyloarthritis *erfasst [18]. Art und Umfang von PTM und NMTM/RTL sowie deren Auswirkungen auf berufliche und persönliche Lebensbereiche wurden anhand individueller Fragestellungen für axSpA-Patienten erhoben.

### Statistik

Die Daten wurden vorrangig deskriptiv ausgewertet unter Verwendung von Chi-Quadrat-Test, t‑Test und nicht-parametrischem Mann-Whitney-U-Test und dem Standardsignifikanzkriterium α = 0,05. Die Auswertung erfolgte mithilfe des Programms SPSS Version 27.0 [12].

## Ergebnisse

### Demografie, Klinik, Funktionsfähigkeit und Teilhabeeinschränkungen

**Abb. 1:**
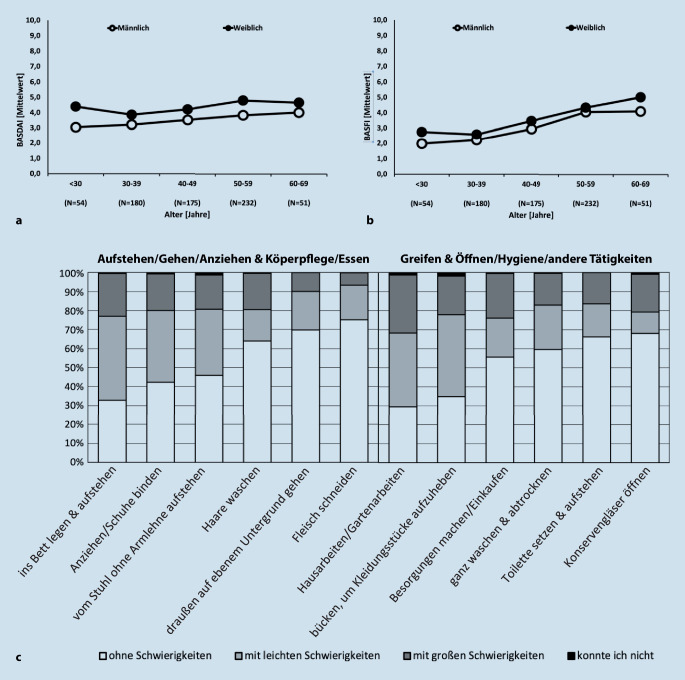
**a** Krankheitsaktivität nach BASDAI über die verschiedenen Altersgruppen. **b** Funktionalität nach BASFI über die verschiedenen Altersgruppen. **c** HAQ-DI: Teilhabeeinschränkungen nach Funktionsbereich und Schwierigkeitsgrad (*N* = 770)

Es wurden die Datensätze von den 770 Patient*innen der Gesamtkohorte untersucht [12]. Wesentliche demografische und klinische Daten sind in der Tab. 1 zusammengefasst. Der Anteil der Männer lag bei 62 %, und das durchschnittliche Alter lag bei 47 (±12,5) Jahren. Als führendes Symptom wurden Rückenschmerzen (88 %) angegeben (Tab. 1). Die mittlere Krankheitsaktivität lag bei einem BASDAI von 3,9 (±2,2). Fast die Hälfte der Patient*innen (47 %) hatte einen BASDAI ≥ 4. Die Abb. 1a zeigt die Höhe des BASDAI bei Männern und Frauen in den verschiedenen Altersgruppen.

**Tab. 1:** Patientencharakteristika, nichtmedikamentöse Therapiemaßnahmen (NMTM) und rehabilitationsmedizinische Therapieleistungen (RTL)

Patientencharakteristika	Gesamt *N* = 770	Mitgliedschaft in einer Selbsthilfeorganisation		
		Ja *n* = 100 (13,0 %)	Nein *n* = 670 (87,0 %)	*p*-Wert
Alter in Jahren	47,1 (±12,5)	52,8 (±9,9)	46,2 (±12,6)	**<** **0,001**
Männer, *n* (%)	474 (61,6)	55 (55,0)	419 (62,5)	0,148
BMI (kg/m^2^)	28,0 (±11,6)	27,5 (±5,0)	28,0 (±12,3)	0,673
axSpA-Erkrankungsdauer in Jahren	13,4 (±11,9)	16,3 (±13,8)	13,0 (±11,5)	**0,026**
Universitätsabschluss, *n *(%)	208 (27,0)	38 (38,0)	170 (25,4)	**0,008**
Vollzeit berufstätig, *n* (%)	458 (59,5)	49 (49,0)	409 (61,0)	0,063
Aufgrund axSpA arbeitsunfähig, *n* (%)	120 (15,6)	15 (15,0)	105 (15,7)	0,980
Dauer der Arbeitsunfähigkeit aufgrund axSpA in Jahren	5,6 (±7,9)	5,6 (6,8)	5,6 (8,1)	0,877
Behinderungsgrad erhalten, *n* (%)	419 (54,4)	75 (75,0)	344 (51,3)	**<** **0,001**
Grad der Behinderung (0–100)	45,9 (±17,1)	49,2 (19,4)	45,2 (16,4)	0,153
Fühlt sich ausreichend über die Krankheit aufgeklärt, *n* (%)	683 (88,7)	90 (90,0)	593 (88,5)	0,660
Rückenschmerzen	674 (87,5)	95 (95,0)	579 (86,4)	**0,024**
Arthritis	381 (49,5)	48 (48,0)	333 (49,7)	0,833
Enthesitis	179 (23,2)	39 (39,0)	140 (20,9)	**<** **0,001**
Daktylitis	109 (14,2)	20 (20,0)	89 (13,3)	0,100
Uveitis	105 (13,6)	16 (16,0)	89 (13,3)	0,560
Psoriasis	102 (13,2)	20 (20,0)	82 (12,2)	**0,048**
Chronisch entzündliche Darmerkrankung	62 (8,1)	5 (5,0)	57 (8,5)	0,315
ASAS-HI, 0–17	6,6 (±3,8)	7,4 (±3,4)	6,4 (±3,9)	**0,015**
– ASAS-HI ≥ 5, *n* (%)	529 (68,7)	82 (82,0)	447 (66,7)	**0,002**
– ASAS-HI ≥ 12, *n* (%)	82 (10,6)	11 (11,0)	82 (10,6)	0,903
BASDAI, 0–10	3,9 (±2,2)	4,3 (±1,9)	3,8 (±2,2)	**0,032**
– BASDAI ≥ 4, *n* (%)	362 (47,0)	56 (56,0)	306 (45,7)	0,068
BASFI, 0–10	3,4 (±2,5)	4,0 (±2,2)	3,3 (±2,5)	**0,010**
– BASFI < 2, *n* (%)	364 (47,3)	19 (19,0)	245 (36,6)	**<** **0,001**
– BASFI ≥ 2–3,9, *n* (%)	214 (27,8)	35 (35,0)	179 (26,7)	0,108
– BASFI ≥ 4–5,9, *n* (%)	148 (19,2)	22 (22,0)	126 (18,8)	0,535
– BASFI ≥ 6, *n* (%)	144 (18,7)	24 (24,0)	120 (17,0)	0,187
Aktuelle med. Therapie, *n* (%)				
– bDMARD	396 (51,4)	59 (59,0)	337 (50,3)	0,104
– NSAR	394 (51,2)	54 (54,0)	340 (50,8)	0,617
– csDMARD	114 (14,8)	12 (12,0)	102 (15,2)	0,487
WPAI^a^				
– Absentismus	10,6 (±26,2)	8,3 (±21,0)	10,9 (±26,9)	0,888
– Präsentismus	32,6 (±25,7)	38,2 (±24,5)	31,8 (±25,7)	**0,049**
– Gesamteinschränkung	37,2 (±29,6)	40,2 (±27,2)	36,8 (±29,9)	0,381
– Aktivitätsbeeinträchtigung	41,6 (±26,1)	46,5 (±21,8)	40,8 (±26,6)	**0,020**
Auf RTL vom Arzt aufmerksam gemacht worden, *n* (%)	555 (72,1)	74 (74,0)	481 (72,0)	0,734
Jemals eine RTL erhalten, *n* (%)	451 (58,6)	80 (80,0)	371 (55,4)	**<** **0,001**
Betreute NMTM-Gruppe^b^, *n* (%)	293 (38,1)	57 (57,0)	236 (35,2)	**<** **0,001**
Regelmäßiges körperliches Training^c^, *n* (%)	660 (85,7)	90 (90,0)	570 (85,1)	0,189
**RTL durchgeführt, *n* (%)**				
– Ambulant	88 (11,4)	13 (13,0)	75 (11,2)	0,718
– Stationär	310 (40,3)	55 (55,0)	255 (38,1)	**0,002**
– Amb./Stat.	52 (6,8)	12 (12,0)	40 (6,0)	**0,043**
**Letzte RTL durchgeführt vor Jahren, MW (±SD)**				
– Ambulant	5,3 (±8,0)	4,1 (±5,9)	5,6 (±8,3)	0,137
– Stationär	6,9 (±8,1)	4,3 (±4,5)	7,6 (±8,7)	**0,002**
**NMTM, *n* (%)**				
– Physiotherapie/Krankengymnastik	654 (84,9)	93 (93,0)	561 (83,7)	**0,023**
– Massage/manuelle Therapie	383 (49,7)	62 (62,0)	321 (47,9)	**0,012**
– Wärmetherapie	222 (28,8)	42 (42,0)	180 (26,9)	**0,003**
– Rehabilitationssport	209 (27,1)	29 (29,0)	180 (26,9)	0,744
– Gerätetraining/medizinische Trainingstherapie	183 (23,8)	36 (36,0)	147 (21,9)	**0,003**
– Angeleitete Gruppentherapie zum Bewegungstraining	177 (23,0)	41 (41,0)	136 (20,3)	**<** **0,001**
– Bewegungsbad	166 (21,6)	39 (39,0)	127 (19,0)	**<** **0,001**
– Funktionstraining	143 (18,6)	43 (43,0)	100 (14,9)	**<** **0,001**
– Elektrotherapie	115 (14,9)	26 (26,0)	89 (13,3)	**0,001**
– Ergotherapie	99 (12,9)	23 (23,0)	76 (11,3)	**0,002**
– Kältetherapie	87 (11,3)	19 (19,0)	68 (10,2)	**0,015**
– Ultraschalltherapie	62 (8,1)	18 (18,0)	44 (6,6)	**<** **0,001**
– Lymphdrainage	41 (5,3)	8 (8,0)	33 (4,9)	**0,299**
– Es wurden bisher keine Therapien verordnet	76 (9,9)	3 (3,0)	73 (10,9)	**0,022**

*ASAS-HI* Assessment of SpondyloArthritis International Society-Health Index, *axSpA* axiale Spondyloarthritis, *BASDAI* Bath Ankylosing Spondylitis Disease Activity Index, *BASFI* Bath Ankylosing Spondylitis Functional Index, *BMI* Body Mass Index, *csDMARD* „conventional synthetic disease modifying antirheumatic drug“, *kg* Kilogramm, *m*^*2*^ Quadratmeter, *MW* Mittelwert, *n* Anzahl Patienten, *NSAR* nichtsteroidale Antirheumatika, *SD* Standardabweichung, *WPAI* Work Productivity and Activity Impairment, *NMTM* nichtmedikamentöse Therapiemaßnahme, *RTL* rehabilitationsmedizinische Therapieleistung, *SD* Standardabweichung

Mittelwert (±STABW), sofern nicht anders gekennzeichnet

^a^Analysen zum WPAI beziehen sich auf aktuell erwerbstätige Patienten (*N* = 590)

^b^Rehabilitationssport/Funktionstraining oder beides

^c^Regelmäßiges körperliches Training im Rahmen der axSpA Behandlung

Sowohl die körperliche als auch die globale Funktionsfähigkeit war eingeschränkt (Tab. 1 und Abb. 1c). Die körperliche Funktionseinschränkung gemäß BASFI lag im Durchschnitt bei 3,4 (±2,5). Bei 38 % Patienten (*N* = 292) lag der BASFI bei 4 oder höher. Die Abb. 1b zeigt die Höhe des BASFI bei Männern und Frauen in verschiedenen Altersgruppen. Für die Tätigkeit „ins Bett legen und aufstehen“ berichteten 23 % der Patienten, große Schwierigkeiten dabei zu haben oder diese Tätigkeit nicht ohne Hilfe durchführen zu können, und für die komplexeren Tätigkeiten „Hausarbeiten/Gartenarbeiten“ gaben 32 % der Patienten an, diese Tätigkeit nicht oder nur unter großen Schwierigkeiten durchführen zu können (Abb. 1c). Bei der Tätigkeit „Besorgungen machen und einkaufen“ konnten 24 % diese Tätigkeit nicht oder nur unter großen Schwierigkeiten durchführen. Der ASAS-HI lag in der Kohorte im Durchschnitt bei 6,6 (±3,8) (Tab. 1).

Das Ausmaß der Einschränkungen der beruflichen Teilhabe wurde kürzlich bereits ausführlich publiziert [12] und ist in Tab. 1 noch einmal kurz zusammengefasst. Bei 419 Patienten (54 %) wurde ein Grad der Behinderung (GdB) von durchschnittlich 45,9 (±17,1) festgestellt. Mehr als die Hälfte dieser Patienten (53 %) gaben einen GdB von 50 oder höher an.

### Medikamentöse Therapiemaßnahmen (MTM)

**Abb. 2:**
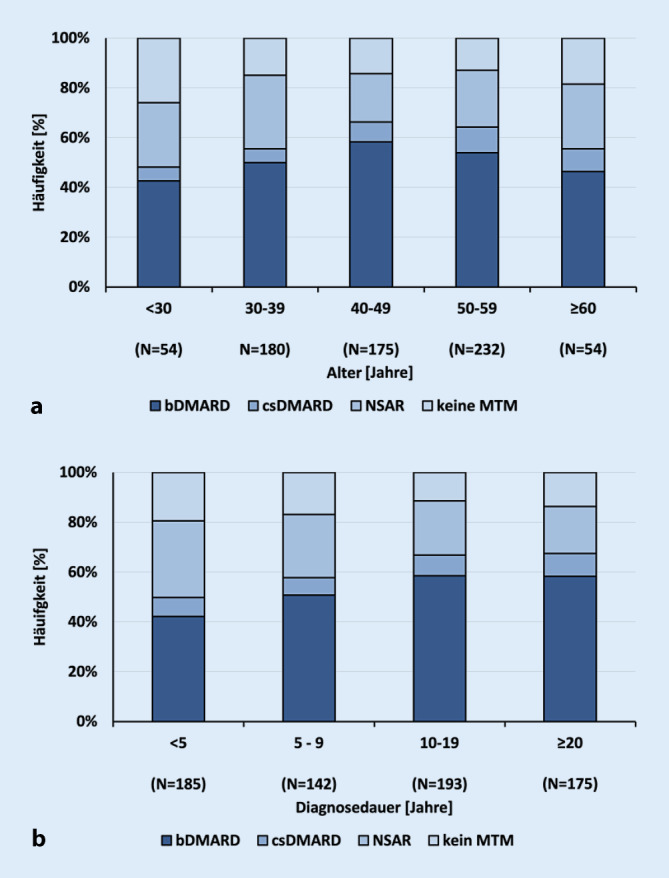
Medikamentöse Therapiemaßnahmen (MTM) nach Alter (**a**) und Diagnosedauer (**b**)

Über 80 % der Patienten erhielten eine MTM. Je nach Alter und Erkrankungsdauer variierte die Häufigkeit innerhalb der Medikationsgruppen (Abb. 2). bDMARDs wurden zum Zeitpunkt der Befragung von 51 % der Patienten angewendet. Der Einsatz der unterschiedlichen MTM abhängig vom Alter und der Diagnosedauer ist in Abb. 2 dargestellt.

### Nichtmedikamentöse Therapiemaßnahmen (NMTM)

**Abb. 3:**
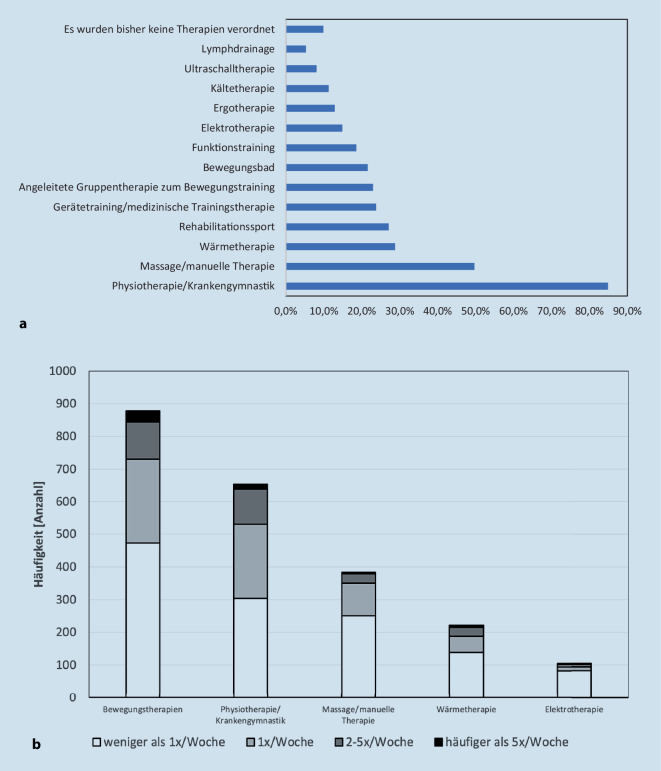
Nichtmedikamentöse Therapiemaßnahmen (NMTM). **a** Bewegungstherapeutische, physiotherapeutische und physikalische NMTM nach Häufigkeit der Angaben (694 Patienten erhielten 2541 Anwendungen). **b** Intensität pro Woche der aktuell durchgeführten NMTM in den 5 häufigsten Gruppen. Die verschiedenen Maßnahmen mit bewegungstherapeutischem Schwerpunkt wurden zusammengeführt und die Intensität der häufigsten angewendeten Maßnahmen dargestellt

Physiotherapie/Krankengymnastik erreichte mit 85 % als Einzeltherapiemaßnahme den höchsten Anteil bei den NMTM (Abb. 3a). Fasst man allerdings die verschiedenen bewegungstherapeutischen Maßnahmen, welche in Deutschland aufgrund verschiedener Vorgaben auf unterschiedliche Bereiche untergliedert sind (Rehabilitationssport, angeleitete Gruppentherapie zum Bewegungstraining, Funktionstraining) zusammen, stellen sie den größten Anteil der Anwendungen dar (Abb. 3). Bewegungstherapeutische Maßnahmen wurden bei 54 % der Patient*innen weniger als 1‑mal pro Woche und bei 29 % 1‑mal pro Woche durchgeführt (Abb. 3).

Es gaben 660 Patient*innen (86 %) an, sich regelmäßig zu bewegen, um die Erkrankung gezielt zu verbessern (Tab. 1). Als Maßnahmen wurden in 511 Fällen (52 %) häusliche Übungen in Eigenregie angegeben. Sport in einem Fitnessstudio wurde von 138 Patient*innen (14 %) und Vereinssport von 67 (7 %) angegeben (Mehrfachnennungen waren möglich). Eine individuelle Sportart wurde von 266 Patient*innen (27 %) betrieben, am häufigsten Radsport (*N* = 96), Wandern/Walking/Gehen (*N* = 96), Ausdauersport (*N* = 94) und Gymnastik (*N* = 41).

### Gebrauch von Hilfsmitteln und personelle Unterstützung

**Abb. 4:**
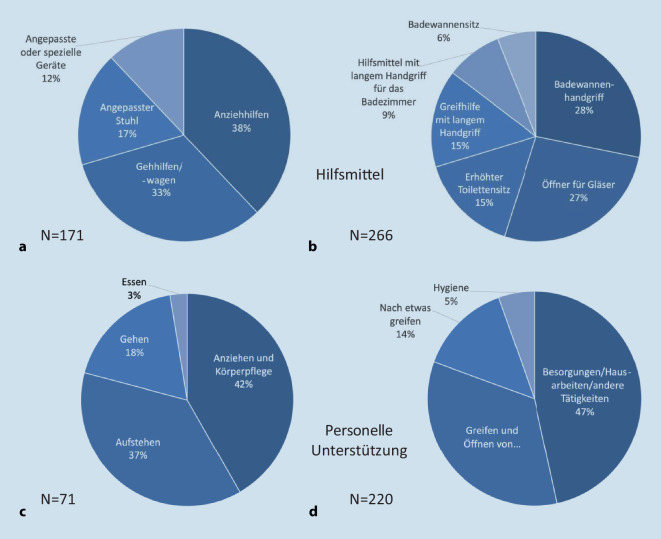
Hilfsmittel und personelle Unterstützung (Angaben nach HAQ). **a**, **b** Benutzte Hilfsmittel. **c**, **d** Personelle Unterstützung

Es benutzten 22 % der Patienten Hilfsmittel für die Tätigkeiten „Aufstehen/Gehen/Anziehen & Körperpflege/Essen“ und 35 % Hilfsmittel für die Tätigkeiten „Greifen und Öffnen/Hygiene/andere Tätigkeiten“. Als häufigste Hilfsmittel wurden Anziehhilfen (37 %) und Badewannengriff (28 %) sowie Öffner für Gläser, die schon einmal geöffnet waren (27 %), genannt (Abb. 4a, b).

Eine Unterstützung durch eine weitere Person erfolgte in dem Bereich „Aufstehen/Gehen/Anziehen & Körperpflege/Essen“ bei 9,2 % (*N* = 71) und in dem Bereich „Greifen und Öffnen/Hygiene/andere Tätigkeiten“ bei 28,6 % der Patient*innen (*N* = 220). Die Unterstützungsmaßnahmen (Mehrfachangaben waren möglich) sind in den Abb. 4c, d dargestellt.

### Rehabilitationsmedizinische Therapieleistungen (RTL)

**Abb. 5:**
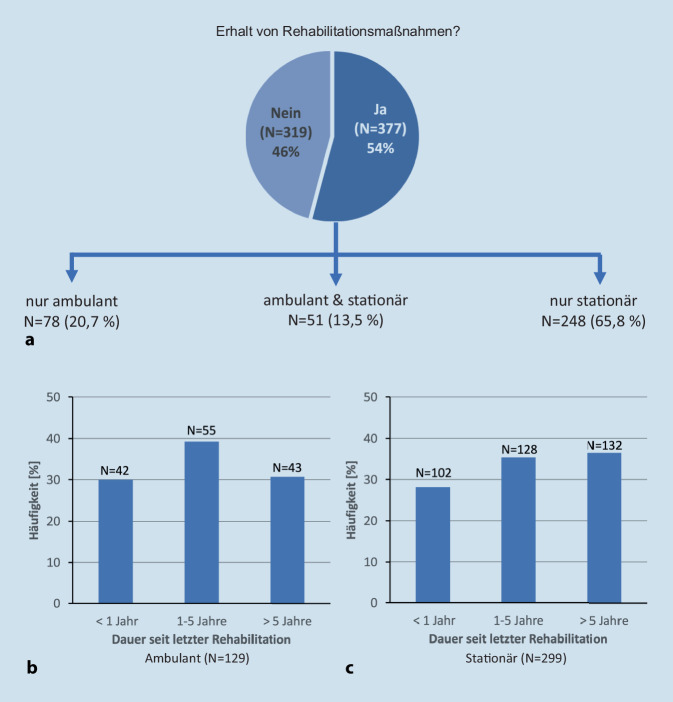
Rehabilitationsmedizinische Therapieleistungen (RTL) (*N* = 696*). **a** Erhalt von ambulanten und/oder stationären Therapieleistungen. **b** Dauer seit letzter ambulanter Rehabilitation. **c** Dauer seit letzter stationärer Rehabilitation. *74 Patient*innen, welche aktuell eine rehabilitationsmedizinische Maßnahme erhielten, wurden aus dieser Analyse ausgeschlossen

Es wurden 72 % der Patient*innen von ihrem behandelnden Arzt auf die Möglichkeit der Teilnahme an einer RTL aufmerksam gemacht. Von den 696 Patient*innen, welche nicht im Rahmen einer Rehabilitationsmaßnahme befragt wurden, gaben 54 % an, jemals eine ambulante oder stationäre RTL erhalten zu haben (Abb. 5). Von den an der Studie teilnehmenden 770 Patient*innen nahmen 74 Patient*innen (9,6 %) aktuell an einer rehabilitationsmedizinischen Maßnahme teil, davon befanden sich 11 Patient*innen in einer ambulanten und 63 Patient*innen in einer stationären Rehabilitationsmaßnahme. Die letzte ambulante RTL lag im Durchschnitt 5,3 (±8,0) Jahre zurück, und die letzte stationäre RTL lag 6,9 (±8,1) Jahre zurück. Bei 31 % der Patient*innen, die angegeben hatten, jemals eine ambulante RTL erhalten zu haben, und bei 37 % der Patient*innen, die angegeben hatten, jemals eine stationäre RTL erhalten zu haben, lag die letzte ambulante bzw. stationäre RTL mehr als 5 Jahre zurück (Abb. 5).

### Mitgliedschaft in einer Selbsthilfeorganisation (SHO)

Es waren 13 % der Patient*innen Mitglied in einer SHO (Tab. 1). Diese waren älter und hatten einen höheren Bildungsgrad (Universitätsabschluss). SHO-Mitglieder hatten eine höhere Krankheitsaktivität (BASDAI), eine stärkere funktionelle Beeinträchtigung (BASFI) und eine geringere globale Funktionsfähigkeit (ASAS-HI) im Vergleich zu Nichtmitgliedern. Nach WPAI gab es in den meisten Domänen keinen signifikanten Unterschied zwischen Mitgliedern und Nichtmitgliedern einer SHO mit Ausnahme von Präsentismus und Aktivitätsbeeinträchtigung (Tab. 1).

Statistisch signifikant mehr Mitglieder einer SHO erhielten jemals eine RTL und hatten nach eigenen Angaben signifikant mehr betreute NMTM von ihrem behandelnden Arzt verschrieben bekommen im Vergleich zu Nichtmitgliedern.

## Diskussion

Die demografischen und klinischen Daten entsprechen den zu erwartenden Zahlen einer Querschnittuntersuchung von Patient*innen mit axSpA in Deutschland [19]. Wir sahen verschiedene Einschränkungen der Alltagsteilhabe mittels des validierten HAQ-DI. Unsere Daten zeigten ein breites Spektrum an Beeinträchtigungen. Insbesondere Tätigkeiten, welche eine größere Beweglichkeit der Wirbelsäule und eine stärkere Beteiligung der Rumpfmuskulatur erfordern (wie z. B. „ins Bett legen und Aufstehen“ oder „bücken, um Kleidungsstücke aufzuheben“), gingen mit deutlich höheren Beeinträchtigungen einher als andere Tätigkeiten und entsprachen somit dem zu erwartenden Befundspektrum bei der axSpA (Abb. 1c). Entsprechend fanden wir auch höhere Beeinträchtigungen bei komplexeren Tätigkeiten (wie z. B. „Hausarbeiten/Gartenarbeiten“). Dieses Bild spiegelt sich ebenfalls in den Angaben bezüglich der benutzten Hilfsmittel oder der persönlichen Unterstützung wider (Abb. 4). Zusammengefasst zeigten unsere Daten ein hohes Maß an Teilhabeeinschränkungen, die im Einklang stehen mit den von uns zuvor beobachteten Einschränkungen der beruflichen Teilhabe [12].

Bezüglich der MTM benutzten nur 51 % der Patienten bDMARDs. Bei den verschiedenen Altersgruppen fand sich die höchste aktuelle Verordnungshäufigkeit zwischen dem 40. und 49. Lebensjahr, während in den jüngeren und älteren Altersgruppen die Verordnungshäufigkeit niedriger war (Abb. 2a). Bei längerer Krankheitsdauer stieg die Verordnungshäufigkeit von bDMARDs kontinuierlich an (Abb. 2b). Allerdings fand sich ein Anteil von fast 40 % der Patienten, welche noch nie mit einem bDMARD behandelt wurden.

Bei den NMTM beobachteten wir ein breites Spektrum an angewandten Maßnahmen (Abb. 3a). Als Einzelmaßnahme hat Physiotherapie/Krankengymnastik mit 85 % den höchsten Anteil. Die bewegungstherapeutischen Maßnahmen als Gruppe stellen zwar den größten Anteil der Anwendungen, wurden aber von über 80 % der Patient*innen nur in einer unzureichenden Intensität (weniger als 1‑mal die Woche oder 1‑mal pro Woche) durchgeführt (Abb. 3b). Wir fanden insgesamt einen hohen Anteil an sportlicher Aktivität in verschiedenen Formen, wobei intensivere Sportarten wie Vereinssport oder Fitnesstraining in einem Studio eher selten waren. Dieses deckt sich mit früheren Beobachtungen aus Deutschland, welche grundsätzlich ein hohes Bewusstsein für die Sinnhaftigkeit von sportlichen Aktivitäten bei Patient*innen mit axSpA feststellten [20]. Es zeigt aber auch, dass insbesondere Maßnahmen von sportlichen Aktivitäten unter Anleitung, wie sie von den Leitlinien empfohlen werden [4], eher seltener durchgeführt werden.

Obwohl fast 55 % der Patient*innen angaben, jemals eine RTL erhalten zu haben (Abb. 5a), lag diese bei etwa einem Drittel mehr als 5 Jahre zurück (Abb. 5b, c). Nach den vorliegenden Daten werden die zur Verfügung stehenden therapeutischen Maßnahmen und Ressourcen häufig nur in einem geringen Maße und/oder in niedriger Intensität wahrgenommen. Die Ursachen hierfür sollten näher untersucht werden.

Nur 13 % der Patient*innen waren Mitglied in einer SHO. Interessanterweise war die Mitgliedschaft in einer SHO mit einer höheren Anzahl von NMTM und einer höheren Nutzung von RTL verbunden. Die Daten belegen eine bedeutende Rolle von SHO bei der Umsetzung leitliniengestützter Behandlungen sowie eine Verbesserung der Selbstmanagementstrategien, die einen günstigen Einfluss auf die Teilhabe der Patient*innen haben können [6, 14].

Zu den möglichen Ursachen der verminderten Inanspruchnahme von NMTM, RTL und SHO könnten Informationsdefizite zum einen von Patient*innen, aber auch von Behandler*innen zählen. Dieses könnte daran liegen, dass in der Vergangenheit ein starker Fokus in der Behandlung berechtigterweise auf medikamentösen Therapiemaßnahmen lag, während NMTM erst in der jüngeren Vergangenheit in Leitlinien verstärkt berücksichtigt wurden. Die Stärken dieser Studie liegen in der hohen Teilnehmerzahl sowie in der elektronischen Datenerhebung und dem externen Monitoring der Zentren zur Verifizierung der Diagnose. Fehlende Werte und Dateneingabefehler konnten durch automatische Abfragen im elektronischen Fragebogen weitestgehend limitiert werden. Damit wurde eine hohe Datenvollständigkeit und -qualität erreicht. Die Schwächen der Studie liegen v. a. am Querschnittcharakter des Studiendesigns und den selbsterstellten Fragen, denen keine Validierungsstudien zugrunde lagen. Damit konnten intraindividuelle Entwicklungsverläufe oder Veränderungen in Bezug auf bestimmte unabhängige Variablen nicht erfasst werden. Die Daten dieser Studie liefern jedoch sehr wertvolle Hinweise hinsichtlich des Einflusses von Selbsthilfegruppen auf die Nutzung der aktuell verfügbaren Therapie- und Rehabilitationsmaßnahmen zur Behandlung der axSpA.

## Fazit für die Praxis

- Teilhabeeinschränkungen bei der axSpA sind vorrangig durch die Einschränkungen der Rumpfbeweglichkeit begründet.
- Trotz deutlich verbesserter Behandlungsstrategien bei der axSpA finden sich weiterhin nur unzureichende Therapieergebnisse in den unterschiedlichen Domänen.
- Die zur Verfügung stehenden therapeutischen Maßnahmen und Gesundheitsressourcen werden häufig nur in einem geringen Maße wahrgenommen.
- Die Mitgliedschaft in einer Selbsthilfeorganisation führt zu einer höheren Nutzung von nichtmedikamentösen Therapiemaßnahmen und Rehabilitationsleistungen.
- Mitglieder von Selbsthilfeorganisationen haben häufiger einen Hochschulabschluss, sind älter und zeigen mehr Krankheitssymptome als axSpA-Patient*innen, die nicht Mitglied in einer Selbsthilfegruppe sind.
